# Hypovolemia and reduced hemoglobin mass in patients with heart failure and preserved ejection fraction

**DOI:** 10.14814/phy2.14222

**Published:** 2019-11-13

**Authors:** David Montero, Thomas Haider, Jens Barthelmes, Jens P. Goetze, Silviya Cantatore, Isabella Sudano, Frank Ruschitzka, Andreas J. Flammer

**Affiliations:** ^1^ University Heart Center University Hospital Zurich Zurich Switzerland; ^2^ Institute of Physiology University of Zurich Zurich Switzerland; ^3^ Department of Clinical Biochemistry, Rigshospitalet University of Copenhagen Copenhagen Denmark

**Keywords:** Blood volume, erythropoietin, heart failure with preserved ejection fraction, hemoglobin mass

## Abstract

A fundamental tenet of heart failure (HF) pathophysiology hinges on a propensity for fluid retention leading to blood volume (BV) expansion and hemodilution. Whether this can be applied to heart failure patients with preserved ejection fraction (HFpEF) remains uncertain. The present study sought to determine BV status and key hormones regulating fluid homeostasis and erythropoiesis in HFpEF patients. BV and hemoglobin mass (Hb_mass_) were determined with high‐precision, automated carbon monoxide (CO) rebreathing in 20 stable HFpEF patients (71.5 ± 7.3 years, left ventricular ejection fraction = 55.7 ± 4.0%) and 15 healthy age‐ and sex‐matched control individuals. Additional measurements comprised key circulating BV‐regulating hormones such as pro‐atrial natriuretic peptide (proANP), copeptin, aldosterone and erythropoietin (EPO), as well as central hemodynamics and arterial stiffness via carotid–femoral pulse wave velocity (PWV). Carotid–femoral PWV was increased (+20%) in HFpEF patients versus control individuals. With respect to hematological variables, plasma volume (PV) did not differ between groups, whereas BV was decreased (−14%) in HFpEF patients. In consonance with the hypovolemic status, Hb_mass_ was reduced (−27%) in HFpEF patients, despite they presented more than a twofold elevation of circulating EPO (+119%). Plasma concentrations of BV‐regulating hormones, including proANP (+106%), copeptin (+99%), and aldosterone (+62%), were substantially augmented in HFpEF patients. HFpEF patients may present with hypovolemia and markedly reduced Hb_mass_, underpinned by a generalized overactivation of endocrine systems regulating fluid homeostasis and erythropoiesis. These findings provide a novel perspective on the pathophysiological basis of the HFpEF condition.

## Graphical abstract

HFpEF patients may present with hypovolemia and markedly reduced hemoglobin mass, underpinned by a generalized overactivation of endocrine systems regulating fluid homeostasis and erythropoiesis. These findings provide a novel perspective on the pathophysiological basis of the HFpEF condition.

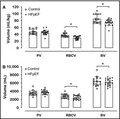

## Introduction

A fundamental principle of cardiovascular physiology refers to the strict dependence of cardiac reserve upon blood volume (BV), which facilitates venous return, ventricular filling, and stroke volume via the Frank–Starling mechanism (Guyton and Hall 2011; Bonne et al. 2014; Montero et al. 2015). Indeed, BV is a tightly regulated fluid varying as little as 4% over several months in healthy individuals, plausibly owing to its central role in maintaining whole body homeostasis (Montero and Flammer 2017; Montero and Lundby 2017). In the presence of a failing heart, acute fluid retention leading to BV expansion is considered a disproportionate compensatory response in the attempt to retrieve cardiac reserve, eventually resulting in clinical congestion (Guyton and Hall 2011). BV may exceed 120 mL·kg^−1^ in untreated heart failure (HF) patients, a ~1.5‐fold increase compared with healthy individuals (Kaplan et al. 1954). In contrast, reduced BVs have been reported in stable HF patients treated with standard pharmacotherapy, including diuretics, after congestion has been alleviated (Anand et al. 1989; Feigenbaum et al. 2000; Bonfils et al. 2010).

Finely tailored medication may be required to sustain euvolemia in HF patients, a challenging task for clinicians since the quantitative measurement of BV is not yet established in the clinical arena (Miller 2016; Ellison and Felker 2017). This uncertainty may particularly affect the hemodynamic stability of HF patients with preserved ejection fraction (HFpEF), a condition characterized by cardiovascular stiffening, limited pressure gradient propelling venous return to the heart, and severe exercise intolerance as a chronic symptom (Schwartzenberg et al. 2012; Montero and Flammer 2017). In these patients, small randomized controlled trials of diuretic withdrawal have shown improvements in ventricular filling and orthostatic tolerance as well as reduced overactivation of the renin–angiotensin–aldosterone system (RAAS), a major endocrine system contributing to the regulation of BV (van Kraaij et al. 2000; 2003). The effects of diuretic withdrawal in HFpEF patients are thus consistent with prior hypovolemia. Yet, relatively little is currently known regarding the BV status of stable HF populations. To date, a single report has indirectly estimated a high prevalence of hypovolemia in HFpEF patients relative to normal BV values derived from anthropometric tables (Abramov et al. 2008). Importantly, a substantial deficit in total hemoglobin mass (Hb_mass_), limiting maximal aerobic capacity (Montero and Flammer 2017; Lundby et al. 2017), may be concealed in hypovolemic HFpEF patients presenting with unaltered or slightly reduced blood hemoglobin (Hb) concentration (Abramov et al. 2008). The combination of hypovolemia and reduced Hb_mass_ could be an overlooked dominant substrate in the pathophysiology of HFpEF. An investigation aimed to characterize BV status and its endocrine regulation, including hormones governing intravascular volumes and Hb_mass_, is warranted in HFpEF patients, but to our knowledge, this has not yet been performed.

The main purpose of the present study was to determine BV, Hb_mass_, and key hormones regulating fluid homeostasis and erythropoiesis in stable HFpEF patients and healthy age‐ and sex‐matched control individuals. We tested the hypothesis that patients with HFpEF will demonstrate hypovolemia and reduced Hb_mass_ in parallel to altered endocrine regulation of BV and erythropoiesis.

## Methods

### Ethical approval

The study was approved by Ethical Commission of Zurich (BASEC‐Nr. 2016‐02167) and conducted in accordance with the Declaration of Helsinki. Prior to the start of the experiments, informed oral and written consents were obtained from all participants.

### Study population

Twenty stable HFpEF patients (left ventricular ejection fraction (LVEF) = 55.7 ± 4.7%, 25% females) were recruited from the HF outpatient clinic of the University Hospital of Zurich. Inclusion criteria comprised: signs and symptoms of HF (Ponikowski et al. 2016), LVEF > 50%, elevated levels of N‐terminal pro‐b‐type natriuretic peptide (NT‐proBNP > 125 pg·mL^−1^), relevant structural/functional cardiac alterations, and no history of iron deficiency (ID) anemia. Healthy age‐ and sex‐matched individuals were recruited from the community and excluded if they had any chronic medical illness, were taking daily prescription medications, had current medical symptoms, or were performing aerobic exercise on a regular basis given its potential effect on BVs and Hb_mass_ (Montero and Flammer 2017, #34).

### Experimental design

All measurements were accomplished within a period spanning from 8:30 am to 11:00 am, after fasting overnight in a quiet room with controlled temperature between 22°C and 24°C. Upon arrival at the laboratory, individuals completed demographic and clinical questionnaires and rested in the supine position for 10 min in order to stabilize cardiovascular and hematological variables. Subsequently, hemodynamics, arterial stiffness, and BV measurements were sequentially performed. Blood and salivary samples were sent for immediate analysis to the Institute of Clinical Chemistry (University Hospital Zurich). Duplicates of blood samples were centrifuged and stored at −80°C for further examination.

### Experimental measures

#### Blood volumes and hemoglobin mass

The method to determine BVs and Hb_mass_ comprised the classic carbon monoxide (CO) rebreathing technique integrated in a fully automated system with a very low typical error of measurement (OpCO, Detalo, Denmark) (Dandanell et al. 2016; Montero et al. 2017). In brief, following supine rest, 2 mL of blood was sampled from an antecubital vein via a 20‐G venflon (BD, USA) and analyzed immediately in duplicate for percent carboxyhemoglobin (%HbCO), Hb concentration, O_2_ saturation (sO_2_), and hematocrit (Hct) with an hemoximeter (ABL800, Radiometer, Denmark). Subsequently, individuals breathed 100% O_2_ for 4 min to flush the nitrogen from the airways. After closing the O_2_ input, a bolus of 1.5 mL kg^−1^ of 99.997% chemically pure CO (CO N47, Air Liquide, France) was administrated into the breathing circuit. Individuals rebreathed this gas mixture for 10 min. Then, an additional 2‐mL blood sample was obtained and analyzed in duplicate as aforementioned. The change in %HbCO is used to calculate Hb_mass_, taking into account the amount of CO that remains in the rebreathing circuit at the end of the procedure. Total red blood cell volume (RBCV), plasma volume (PV), and BV were determined from Hb_mass_, Hb, and Hct (Dandanell et al. 2016; Montero et al. 2017).

#### Hematology

Blood samples (5 mL) from the antecubital vein were collected anaerobically in heparinized glass syringes. Serum creatinine was assessed via the kinetic Jaffe reaction (Hitachi P‐Modular system, Roche Diagnostics, Switzerland). Estimated glomerular filtration rate (eGFR) was calculated using the CKD‐EPI Creatinine Equation (2009) according to the guidelines from the National Kidney Foundation (Inker et al. 2014). Hormones measured in plasma included pro‐atrial natriuretic peptide (proANP), NT‐proBNP, copeptin (a surrogate of vasopressin) (Morgenthaler 2010), erythropoietin (EPO), and aldosterone. Pro‐atrial natriuretic peptide (proANP) was assessed with a mid‐regional assay on a Kryptor Plus platform (Thermo Fisher, Germany) (Hunter et al. 2011), while NT‐proBNP was determined by immunoassay (Elecsys NT‐proBNP, Roche Diagnostics, Switzerland). Copeptin was assessed using an automated immunofluorescent assay (Thermo Fisher, Germany) (Morgenthaler et al. 2008; Balanescu et al. 2011; Roussel et al. 2014). The Human EPO Quantikine IVD ELISA Kit (R&D Systems Inc., USA) was used to measure EPO. The observed/predicted (O/P) EPO ratio was calculated according to the regression equation Log(EPO) = 4.746 − (0.275 × Hb) (Beguin et al. 1993; Opasich et al. 2005; Meer et al. 2008). Aldosterone and salivary cortisol were quantified by means of a competitive enzyme immunoassay (R&D Systems Inc., USA). In addition, in order to control for potential confounding acute alterations in hemoconcentration, albumin levels were assessed with ALB reagent in conjunction with UniCel^®^ DxC 600/800 and Synchron^®^ Systems Multi Calibrator (Beckman Coulter, USA).

#### Hemodynamics

The internal jugular vein (IJV) aspect ratio, a surrogate of central venous pressure (CVP), was determined at the level of the cricoid cartilage using the method described by Keller et al. (2009) In brief, the left IJV was assessed by means of a 7 MHz linear array probe attached to a high‐resolution ultrasound machine (SonixTouch, BK Ultrasound, USA). After obtaining an optimized IJV image, a 20‐sec B‐mode cine loop was obtained and reviewed frame‐by‐frame to identify the largest cross‐sectional area (during expiration), and vessel dimensions were recorded. The IJV height was divided by its width to obtain the aspect ratio. In addition, pulse wave analysis (PWA) was performed on radial artery pressure waveforms (SphygmoCor, AtCor Medical, Australia).

#### Arterial stiffness

Carotid–femoral pulse wave velocity (PWV) was assessed with arterial tonometry (SphygmoCor, AtCor Medical, Australia) according to established guidelines (Van Bortel et al. 2012). Pressure waveforms were acquired at the left common carotid and femoral arteries. To determine carotid–femoral PWV, the difference in the time of pulse arrival between common carotid and femoral arteries from the R‐wave of the electrocardiogram was calculated using the intersecting tangent algorithm. Moreover, carotid artery distensibility, a relatively less established assessment of arterial stiffness, was assessed by means of high‐resolution ultrasound (SonixTouch, BK Ultrasound, USA) (Van Bortel et al. 2002). The distensibility coefficient (DC) was calculated according to the following formula:

$$\text{DC}=\left(2\Delta D\times D+\Delta D^{2}\right)/\left(PP\times D^{2}\right)$$

where *D* is the arterial diameter, ∆*D* is the distension, and PP is the brachial pulse pressure. DC represents the reciprocal value of arterial stiffness (AS).

### Statistical analysis

Statistical analysis was performed using SPSS 22.0 (SPSS, Chicago, IL). Data were tested for normal distribution with the Kolmogorov–Smirnov test and for homogeneity of variances with Levene's test.

Fisher's exact test, independent *t*‐test, and Mann–Whitney *U*‐test were used to compare dichotomous, parametric, and nonparametric data, respectively, between HFpEF patients and control individuals. Data are reported as mean ± SD unless otherwise stated. A two‐tailed *P*‐value less than 0.05 was considered significant.

## Results

### Population characteristics

Baseline characteristics of HFpEF patients and control individuals are reported in Table 1. HFpEF patients presented with increased body mass index (*P* = 0.003) and NT‐proBNP (*P* < 0.001) while eGFR was mildly reduced (*P* = 0.002) compared with control individuals. Venous Hb concentration (*P* = 0.002) and Hct (39.7 ± 5.2 vs. 44.0 ± 1.8%, *P* = 0.002) were also reduced in HFpEF patients. No difference was detected for venous sO_2_ (82.5 ± 11.9 vs. 80.3 ± 8.9%, *P* = 0.547) between groups. The most prevalent comorbidities in HFpEF patients were hypertension (80%), coronary artery disease (55%), and diabetes mellitus (25%). With respect to the medication, beta‐blockers (70%), loop diuretics (60%), and angiotensin‐converting enzyme inhibitors/angiotensin II‐receptor blockers (60%) were common and similarly prevalent in HFpEF patients. Control individuals were healthy and did not take medications.

**Table 1:** Baseline demographic and clinical characteristics.

	Control	HFpEF	P
*n*	15	20	
Age (years)	70.6 ± 5.3	71.5 ± 7.3	0.683
Sex (female/male)	2/13	5/15	0.672
Height (cm)	175.4 ± 7.6	169.6 ± 8.9	0.068
Weight (kg)	75.7 ± 11.5	83.8 ± 17.8	0.133
BMI (kg·m^−2^)	24.7 ± 3.2	29.2 ± 4.4	**0.003**
BSA (m^2^)	1.91 ± 0.17	1.95 ± 0.23	0.609
HR (bpm)	57.9 ± 9.8	62.9 ± 10.3	0.245
SBP (mm Hg)	136.1 ± 14.4	140.4 ± 19.6	0.476
DBP (mm Hg)	80.0 ± 9.0	78.6 ± 9.1	0.653
NT‐proBNP (ng·L^−1^)	78.1 ± 42.9	823.6 ± 725.6	**<0.001**
Fasting glucose (mmol·L^−1^)	5.5 ± 0.4	5.9 ± 1.1	0.458
eGFR (mL·min^−1^)	82.7 ± 9.5	64.0 ± 20.1	**0.002**
Hb (g·dL^−1^)	14.4 ± 0.6	12.9 ± 1.7	**0.002**
Ferritin (*µ*g·L^−1^)	194.9 ± 110.6	150.9 ± 99.9	0.226
Smoking (yes/no)	0/15	3/20	0.244
Comorbidities (%)			
CAD	–	55	
HTN	–	80	
DM	–	25	
Medication (%)			
ACEI/ARB	–	60	
BB	–	70	
Loop DIU	–	60	
Lipid lowering	–	55	

Data are presented as mean ± SD, ratio or %.

*P* < 0.05 (in bold).

ACEI/ARB, angiotensin‐converting enzyme inhibitors or angiotensin II‐receptor blockers; BB, beta‐blockers; BMI, body mass index; BSA, body surface area; CAD, coronary artery disease; DBP, diastolic blood pressure; DM, diabetes mellitus; eGFR, estimated glomerular filtration rate; Hb, hemoglobin; HR, resting heart rate; HTN, hypertension; Loop DIU, loop diuretics; NT‐proBNP, N‐terminal pro‐b‐type natriuretic peptide; SBP, systolic blood pressure.

### Central hemodynamics and arterial stiffness

Table 2 presents hemodynamics and distensibility parameters of elastic arteries. HFpEF patients exhibited augmented IJV aspect ratio, an estimate of CVP, compared with control individuals (*P* = 0.034). Aortic Aix@75 was similar between HFpEF patients and control individuals. Likewise, carotid distensibility did not differ between groups. In contrast, carotid–femoral PWV was increased in HFpEF patients (*P* = 0.047).

**Table 2:** Central hemodynamics and arterial stiffness.

	Control	HFpEF	*P*
Central hemodynamics			
IJV aspect ratio	0.54 ± 0.21	0.71 ± 0.18	**0.034**
Aortic SBP (mm Hg)	131.0 ± 15.4	134.5 ± 17.7	0.546
Aortic DBP (mm Hg)	80.9 ± 6.1	82.4 ± 10.1	0.620
Aortic Aix@75 (%)	23.7 ± 7.2	25.8 ± 8.9	0.464
Arterial stiffness			
Carotid D_dia_ (mm)	7.37 ± 0.83	7.39 ± 1.15	0.963
Carotid D_sys_ (mm)	7.77 ± 0.89	7.72 ± 1.13	0.890
Carotid distensibility (10^3^ kPa^−1^)	2.1 ± 1.2	1.6 ± 0.7	0.182
Carotid–femoral PWV (m·sec^−1^)	8.6 ± 2.1	10.4 ± 2.9	**0.047**

Data are presented as mean ± SD.

*P* < 0.05 (in bold).

Aix@75, augmentation index adjusted by heart rate of 75 bpm; DBP, diastolic blood pressure; D_dia_, diastolic diameter; D_sys_, systolic diameter; PWV, pulse wave velocity; SBP, systolic blood pressure.

### Blood volumes and hemoglobin mass (Hb_mass_)

**Figure 1:**
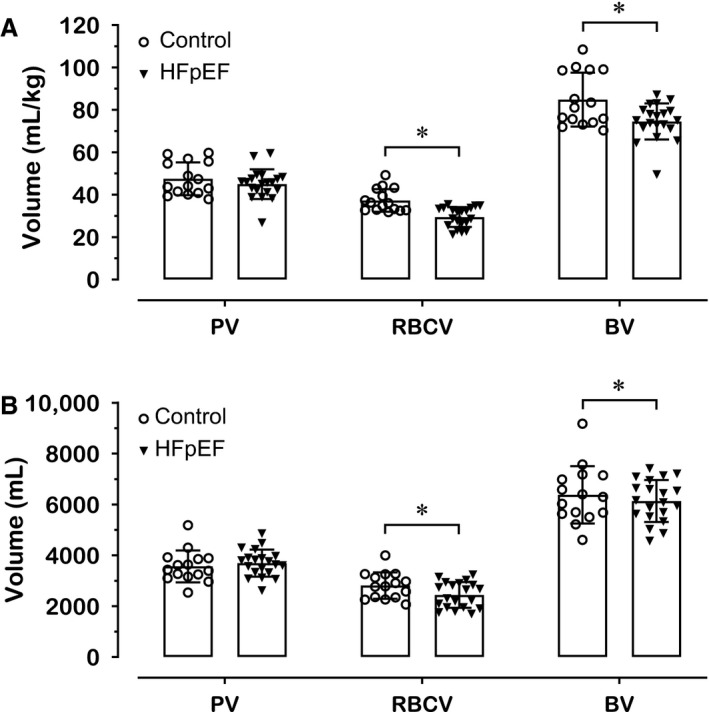
Blood volumes in relative (A) and absolute (B) units in HFpEF patients and control individuals. Data in bars are presented as mean ± SD. *Significantly different (*P* < 0.05) between HFpEF patients and control individuals. BV, blood volume; HFpEF, heart failure with preserved ejection fraction; PV, plasma volume; RBCV, red blood cell volume.

Blood volumes in HFpEF patients and control individuals are displayed in Figure 1. Both groups had comparable PV (45.0 ± 7.0 vs. 47.5 ± 7.7 mL·kg^−1^, *P* = 0.324), whereas RBCV (29.5 ± 4.7 vs. 37.3 ± 5.4 mL·kg^−1^, *P* < 0.001) and BV (74.5 ± 8.5 vs. 84.8 ± 12.7 mL·kg^−1^, *P* = 0.012) were diminished in HFpEF patients. Similar results were observed for BVs expressed in absolute units (PV: 3692 ± 534 vs. 3567 ± 628 mL, *P* = 0.528; RBCV: 2444 ± 507 vs. 2813 ± 520 mL, *P* = 0.043) (Fig. 1). Consistent with RBCV results, Hb_mass_ was reduced in HFpEF patients in relative (9.6 ± 1.5 vs. 12.2 ± 1.8 g·kg^−1^, *P* < 0.001) and absolute units (796.2 ± 166.2 vs. 917.6 ± 169.5 g, *P* = 0.042).

### Blood volume‐regulating hormones

**Figure 2:**
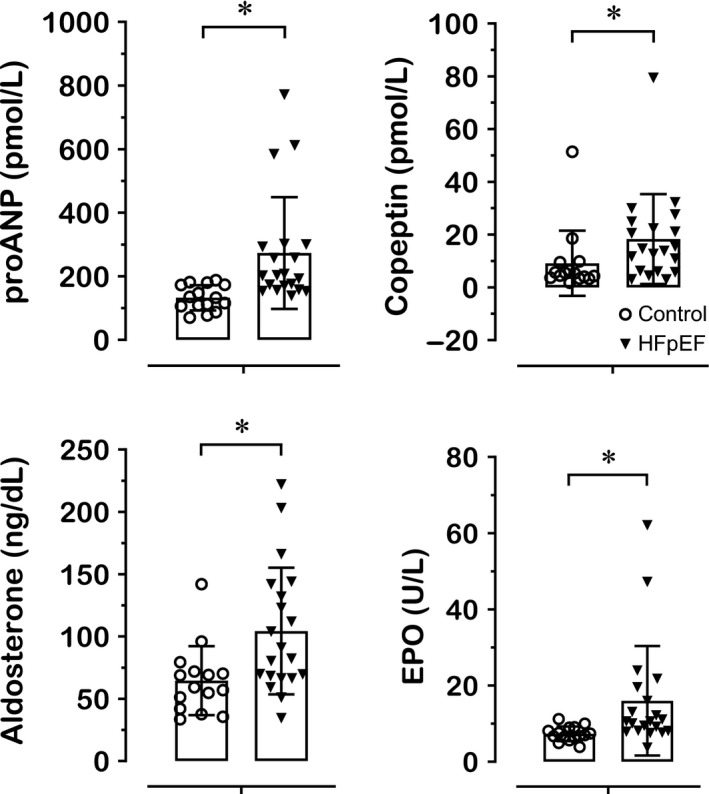
Blood volume‐regulating hormones in HFpEF patients and control individuals. Data in bars are presented as mean ± SD. *Significantly different (*P* < 0.05) between HFpEF patients and control individuals. EPO, erythropoietin; HFpEF, heart failure with preserved ejection fraction; proANP, pro‐atrial natriuretic peptide.

Figure 2 shows BV‐regulating hormones in HFpEF patients and control individuals. Plasma proANP (273.2 ± 39.3 vs. 132.5 ± 10.3 pmol·L^−1^, *P* < 0.001), copeptin (18.3 ± 3.8 vs. 9.2 ± 3.2 pmol·L^−1^, *P* = 0.008), and aldosterone (104.4 ± 11.4 vs. 64.5 ± 7.1 ng·dL^−1^, *P* = 0.011) concentrations were substantially augmented in HFpEF patients. Moreover, HFpEF patients presented more than twofold enhancement of EPO concentration (16.0 ± 3.2 vs. 7.3 ± 0.5 U·L^−1^, *P* < 0.001), which resulted in similar O/P EPO ratio (1.06 ± 0.52 vs. 1.11 ± 0.26, *P* = 0.227) between groups. Albumin levels did not differ between HFpEF patients and control individuals (40.2 ± 3.9 vs. 41.6 ± 1.9 g·L^−1^, *P* = 0.202).

## Discussion

The present study assessed intravascular volumes, Hb_mass_, and endocrine regulation in stable HFpEF patients and healthy age‐ and sex‐matched control individuals. The main findings evidence that HFpEF patients may present with: (1) preserved PV, (2) marked reductions in RBCV and Hb_mass_, resulting in hypovolemia, and (3) augmented levels of BV‐regulating hormones including copeptin, aldosterone, and EPO, denoting the activation of negative feedback mechanisms elicited by hypovolemia.

Fluid retention represents a primary clinical manifestation of decompensation in HF patients (Miller and Mullan 2016). Given the continuous interaction between extracellular fluids, volume expansion concerns both interstitial and intravascular constituents. Hypervolemia entails fundamental alterations of circulatory homeostasis frequently considered as a life‐threatening condition that requires immediate medical attention (Gheorghiade et al. 2013). Once the patient is acutely treated and stabilized, some degree of BV expansion persists at clinical discharge, particularly in HF patients with reduced ejection fraction (HFrEF) (Miller and Mullan 2016). In the short term, pharmacological interventions decreasing BV may improve the functional status of HFrEF patients (Okonko et al. 2019, #121). With chronic treatment, however, HFrEF patients may develop hypovolemia (Anand et al. 1989; Feigenbaum et al. 2000; Bonfils et al. 2010). To date, hypovolemia in HFpEF patients following standard pharmacotherapy has been solely supported by indirect evidence (van Kraaij et al. 2000; 2003; Abramov et al. 2008; Noumi et al. 2011; Montero and Flammer 2017). The current study demonstrates a 14% reduction in BV in stable HFpEF patients compared with control individuals. In absolute terms, BV levels in HFpEF patients were slightly lower compared with those of stable HFrEF patients reported in other studies (Abramov et al. 2008, #61; Miller 2016, #64) As expected, HFpEF patients also presented with increased stiffness of central elastic arteries as determined by augmented carotid–femoral PWV, previously attributed, among other factors, to the exacerbated degradation of elastic fibers in the arterial wall mediated by proteolytic enzymes such as matrix metalloproteinase‐2 and ‐9 (Yasmin et al. 2005; Somleva et al. 2013). With regard to compounding effects of hypovolemia and arterial stiffness, experimental studies indicate that decreased BV impairs ventricular filling, stroke volume, and cardiac output specifically in the HFpEF phenotype, notably in the presence of exacerbated stiffening of the cardiovascular system (Nagano et al. 1997). Loss of central arterial distensibility limits elastic recoil and contributes to ventricular diastolic dysfunction, limiting the gradient for venous return, further constrained by hypovolemia (Luers et al. 2017). In addition, lower central BV unloads arterial and cardiopulmonary baroreceptors (Montero et al. 2016), inducing a compensatory response that enhances sympathetic nerve activity and vasomotor tone (Ryan et al. 2012) as well as activates the RAAS ultimately leading to increased arterial stiffness (Fig. S1) (Rehman et al. 2002). Collectively considered, the apparent hemodynamic stability of HFpEF patients may be sustained by chronic baroreflex‐mediated overactivation of endocrine negative feedback loops attempting to restore euvolemia.

This investigation reveals another intriguing hematological characteristic of stable HFpEF patients. While the high prevalence of anemia, determined from Hb concentration cutoff values, is well established in HF populations (Brucks et al. 2004; Klapholz et al. 2004; Berry et al. 2005), the cause of anemia is commonly attributed to hemodilution owing to presumed PV expansion (Senni et al. 2014). In contrast, the present findings indicate that true (non‐hemodilutional) anemia prevails in HFpEF patients, exhibiting nearly a one‐third reduction in Hb_mass._ This concurs with previous studies reporting a higher prevalence of true anemia in HFpEF compared with HFrEF patients (Abramov et al. 2008, #61; Miller 2016, #64). Of note, Hb_mass_, rather than Hb concentration, is a crucial determinant of maximal oxygen consumption (VO_2max_), a hallmark of exercise tolerance routinely used to ascertain cardiac transplant eligibility in patients with advanced HF (Montero et al. 2015; Montero and Flammer 2017; Lundby et al. 2017). In the absence of PV expansion, Hb_mass_ can be substantially reduced despite the Hb concentration falling within normal limits. The predominant influence of Hb_mass_ on VO_2max_ is explained by the former being intrinsically associated with both hematological (blood oxygen‐carrying capacity) and hemodynamic (BV, ventricular preload) variables determining convective oxygen delivery (Montero et al. 2015; Lundby et al. 2017). Hence, a key underlying cause of severe exercise intolerance, which represents a main chronic symptom of HFpEF (Montero and Lundby, 2017), may have been concealed in this condition.

The question arises as to which mechanisms underlie hypovolemia and reduced Hb_mass_ in HFpEF patients. BV is subjected to endocrine regulation via natriuretic peptides, vasopressin, and the RAAS (Gauer and Henry 1963). These hormones primarily control PV, while EPO governs erythropoiesis and thus RBCV (Lundby and Olsen, 2011). When central baroreceptors sense a decrease in the filling state of the cardiovascular system, afferent signals to the central nervous system prompt the release of vasopressin from the posterior pituitary and enhance efferent sympathetic nervous activity to the kidney stimulating the RAAS hormonal cascade (Montero et al. 2016). Vasopressin and RAAS hormones facilitate PV retention as well as stimulate EPO production (Zivny et al. 1972; Engel and Pagel 1995; Freudenthaler et al. 1999). Herein, HFpEF patients presented with approximately 1.5‐ to 2.5‐fold elevations of copeptin (a proxy for vasopressin), aldosterone, and EPO. A similar increment was observed for proANP, a natriuretic peptide secreted by atrial cells in response to atrial stretch with opposite renal effects to those of vasopressin and RAAS hormones (Goetze et al. 2015). Taking into account the attenuated responsiveness to natriuretic peptides in HF (Hartupee and Mann 2017), augmented vasopressin and aldosterone may contribute to maintain normal PV levels in stable HFpEF patients, most of which are paradoxically treated with loop diuretics to prevent eventual decompensation (Montero and Flammer 2017; Hallow et al. 2018). Parenthetically, the ineffectiveness of neurohormonal antagonism targeting the adrenergic system and RAAS in HFpEF implies that this condition might not involve neurohormonal activation as a fundamental pathophysiologic mechanism (Oghlakian et al. 2011); such alteration could merely be a compensatory response opposing hypovolemia resulting from a deficit in RBCV. With respect to the cause(s) of impaired erythropoiesis in HFpEF patients, the unaltered O/P EPO ratio denotes adequate EPO production, thus suggesting a resistance to the action of EPO in the bone marrow. Among potential culprits, obesity‐related systemic low‐grade inflammation may directly alter bone marrow activity (Redfield, 2016). Erythropoiesis is affected by circulating pro‐inflammatory cytokines as tumor necrosis factor‐alpha interleukin‐1 and interferon‐gamma, which hinder erythroid proliferation and differentiation (Means, 1995; Selleri et al. 1996). Considering the lack of effective treatment for HFpEF (Ponikowski et al. 2016), further research is warranted to determine the role of potential phenotypic modifications of the hematopoietic bone marrow in this population (Anand and Rector 2014). This could disclose novel biological targets, notably for those anemic patients in whom EPO treatment is ineffective or detrimental (Borovka et al. 2013; Swedberg et al. 2013).

### Limitations

There are some limitations to this study that require comment. The recruitment of stable HFpEF patients with preserved functional capacity precludes the generalization of findings to patients with advanced disease, albeit the demographics and clinical characteristics of the HFpEF group closely matched those of systematic HF reviews (Montero et al. 2017). Nonetheless, given the relationship of cardiovascular disease progression with sedentary behavior (Ford and Caspersen, 2012), which inexorably leads to hypovolemia, (Convertino 2007) the reduction in BV and Hb_mass_ might be exacerbated in later stages of the HFpEF condition. Moreover, the narrow range of diuretic doses in our cohort limits the possibility to assess the specific impact of diuretics on PV and hemodynamics (Montero and Flammer 2017). Further studies are required to elucidate the role of prevailing HF therapy including loop diuretics and neurohormonal antagonists on BV status and Hb_mass_ in HFpEF patients. In addition, HFpEF patients may present ID, a prevalent comorbidity associated with severe diastolic dysfunction and decreased exercise capacity (Bekfani et al. 2019, #133). In this respect, experimental studies have demonstrated that ID directly affects human cardiomyocytes, leading to mitochondrial dysfunction and decreased ventricular contractility and relaxation (Hoes et al. 2018, #134). Despite we excluded HFpEF patients with a history of ID anemia and ferritin levels were similar between HFpEF patients and healthy control individuals, the presence of some degree of ID cannot be discarded in our study. Finally, the noninvasive methodology used to estimate CVP, based on the IJV ratio, be regarded as suggestive rather than definitive.

## Conclusion

In stable HFpEF patients, intravascular volume status is characterized by hypovolemia and reduced Hb_mass_, in parallel to a generalized overactivation of hormones regulating fluid homeostasis and erythropoiesis. These data provide key pathophysiological evidence distinguishing a plausibly prevalent phenotype of HFpEF. The implication of these findings for identifying effective therapies in this population remains to be substantiated in future studies.

## Conflict of Interest

None declared.

## Supporting information




**Figure S1.** Potential mechanisms linking hypovolemia with impaired cardiac function.
