# Supplementary figures and images for: Hypovolemia and reduced hemoglobin mass in patients with heart failure and preserved ejection fraction
Source: Physiol Rep. 2019 Nov 13;7(21):e14222. doi: 10.14814/phy2.14222 (PMC6854115; doi:10.14814/phy2.14222)

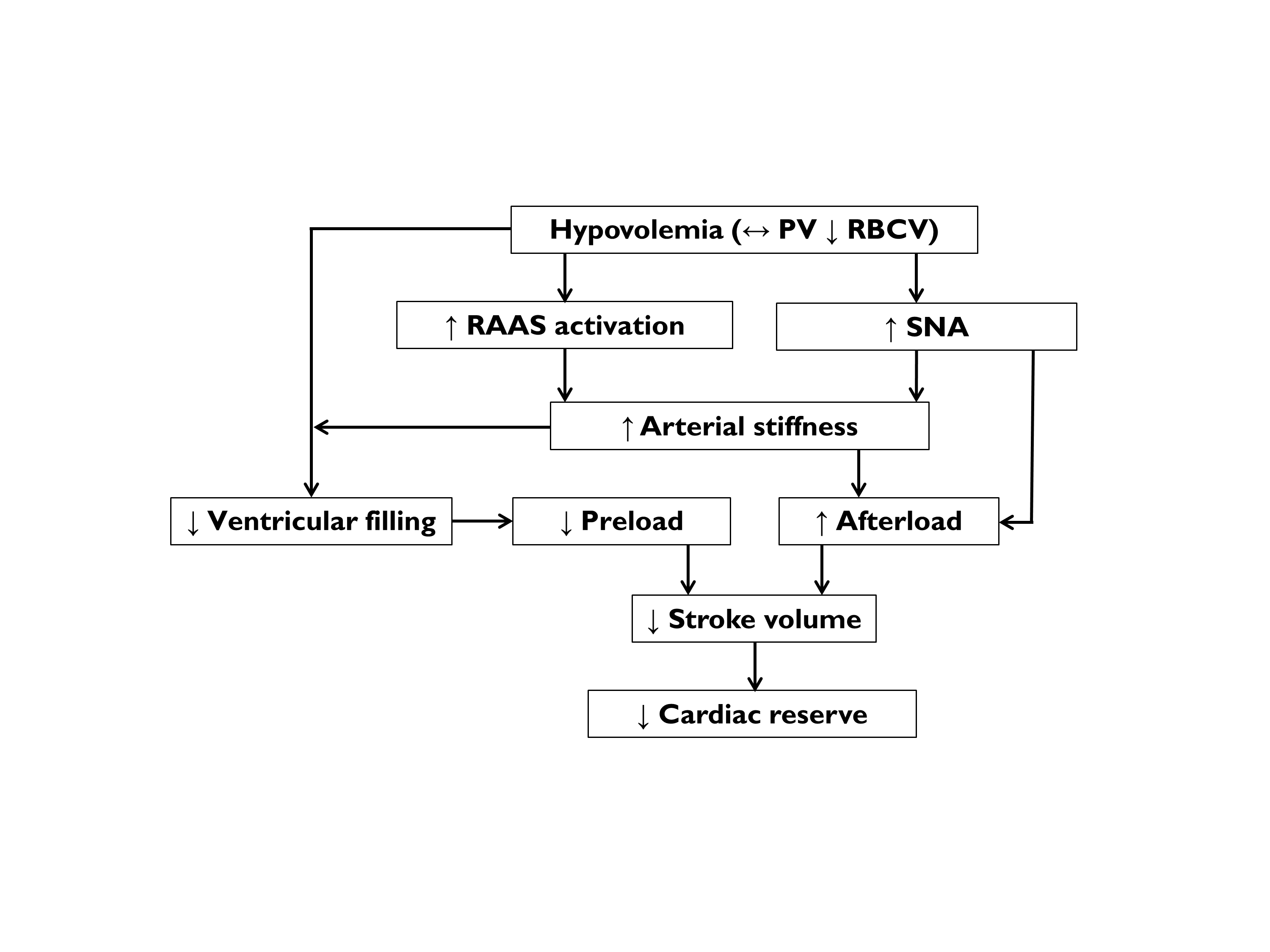

Supplement: Supplementary file 1 — Figure S1. Potential mechanisms linking hypovolemia with impaired cardiac function. [file PHY2-7-e14222-s001.tif]
